# Sperm DNA damage and its role in IVF and ICSI

**DOI:** 10.1186/s12610-016-0043-6

**Published:** 2016-12-05

**Authors:** Phil Vu Bach, Peter N. Schlegel

**Affiliations:** Department of Urology, Weill Cornell Medicine, 525 East 68th Street, Starr 900, New York, NY 10065 USA

**Keywords:** IVF, ICSI, Sperm, DNA fragmentation, SCD, Halo, SCSA, Comet, TUNEL, FIV, ICSI, Spermatozoïde, Fragmentation de l’ADN, Halo, SCSA, Comet, TUNEL

## Abstract

While the semen analysis has traditionally been relied upon to differentiate fertile and infertile men, its utility has been questioned in the current era of assisted reproductive technologies. The desire for more sophisticated diagnostic and predictive tools has led to increased use of sperm DNA damage in the management of male infertility. Despite the availability of numerous assays to measure sperm DNA damage, our understanding of the etiology, measurement, and clinical implications of sperm DNA damage remains incomplete. While the current evidence is fraught with heterogeneity that complicates attempts at comparison and meta-analysis, there does appear to be a role for sperm DNA damage in the development and maintenance of pregnancy in the era of in vitro fertilization (IVF) and intracytoplasmic sperm injection (ICSI). However, as noted by the American Society for Reproductive Medicine, the routine and widespread use of sperm DNA damage testing is not yet supported. Further studies are needed to standardize the measurement of sperm DNA damage and to clarify the exact role of sperm DNA damage within the myriad of other male and female factors contributing to reproductive outcomes in IVF and ICSI.

## Résumé

Si l’analyse de sperme a traditionnellement reposé sur la distinction des hommes féconds des inféconds, son utilité a été remise en question à notre époque d’assistance médicale à la procréation.

Le souhait d’outils diagnostiques et prédictifs plus sophistiqués a mené à une utilisation croissante de l’altération de l’ADN spermatique dans la prise en charge de l’infécondité masculine. Malgré la présence de nombreux tests disponibles pour évaluer l’altération de l’ADN spermatique, notre compréhension de l’étiologie, de la mesure et de des implications cliniques reste incomplète.

Bien que la preuve actuelle soit entachée d’une hétérogénéité qui complique les tentatives de comparaison et de méta-analyses, l’altération de l’ADN semble bien avoir un rôle dans le développement et le maintien de la grossesse à notre époque de fécondation in vitro (FIV) et d’injection intra cytoplasmique d’un spermatozoïde (ICSI). Toutefois, comme l’a fait remarquer l’American Society for Reproductive Medicine, une large utilisation de l’évaluation de l’altération de l’ADN en routine manque encore de support. De futures études sont nécessaires pour la standardisation de la mesure de l’altération de l’ADN et pour élucider le rôle exact de cette altération parmi la myriade des autres facteurs masculins et féminins qui contribuent aux issues reproductives de la FIV et de l’ICSI.

## Background

The semen analysis has traditionally been used to differentiate fertile and infertile men. With the advent of IVF and ICSI, there has been a desire for more sophisticated diagnostic and predictive tools. Sperm DNA damage has been associated with adverse reproductive outcomes and has been increasingly used in the management of male infertility in the era of IVF and ICSI. However, despite the availability of numerous laboratory assays to measure sperm DNA damage, the clinical utility of these tests and their potential roles in the algorithm of male infertility management have yet to be established.

This review seeks to examine the existent literature to discuss our current understanding of sperm DNA damage, the tools available for measuring sperm DNA damage, and their associations with reproductive outcomes after use of IVF and ICSI in an attempt to clarify the role of these tests in the management of male infertility.

## Methods

An extensive computer search of MEDLINE, EMBASE, and PUBMED was performed using combinations of the following search terms: “semen analysis,” spermiogenesis,” “sperm DNA fragmentation,” “sperm DNA damage,” “IVF,” “ICSI,” “outcomes,” “pregnancy,” “Comet,” “TUNEL,” “SCSA,” SCD,” and “Halo.” Reference lists of relevant articles and reviews were also analyzed for further articles. After review of titles and abstracts, a list of relevant articles that discussed semen analysis, sperm DNA fragmentation, and the relationship between semen analysis and/or sperm DNA fragmentation on reproductive outcomes was compiled and included in the review.

### The inadequacy of semen analysis

Along with a complete history and physical exam, semen analysis is the diagnostic pillar for the assessment of male fertility and, thanks to the efforts of the World Health Organization (WHO), has been standardized worldwide. To develop the currently used semen analysis reference ranges, the WHO analyzed semen data from over 4500 men in fourteen countries and selected those that came from 1859 fertile men, defined as those who were able to impregnate their partners within twelve months of unprotected sexual intercourse [1]. From there, the researchers applied a one-sided lower reference limit of the 5^th^ percentile to establish the lower thresholds of a normal semen analysis based on semen volume (1.5 mL), sperm concentration (15 million sperm/mL), total sperm number (39 million sperm/ejaculate), total progressive motility (40 %), morphologically normal sperm (4 %), and sperm vitality (58 %). Unfortunately, while providing a lower thresholds of semen parameters in fertile men, this methodology fails to address the more relevant clinical question of the semen parameters that represent male subfertility or infertility. Indeed, with 7.5 % of men estimated to have fertility problems where a male factor contributes to infertility, even the arbitrary lower reference limit of 5 % for lower levels of abnormal semen parameters is severely flawed.

Efforts to identify specific semen parameters able to discriminate between fertile and subfertile men have mainly used time-to-pregnancy (TTP) as a surrogate for fecundity in couples desiring natural conception. The recent Longitudinal Investigation of Fertility and the Environment (LIFE) study was a prospective, observational cohort study that assessed 501 couples discontinuing contraception with the goal of becoming pregnant with TTP as the primary endpoint. While several semen parameters were associated with differing TTP on univariate analysis, none of the semen parameters reached significance on multivariate analysis [2]. However, increasing male age was associated with decreased fecundity on multivariate analysis (fecundability odds ratio 0.96, 95 % CI 0.93–0.99) [2]. The results from the LIFE study corroborate earlier work attempting to define semen parameters indicative of male infertility. These studies found that while semen parameters were associated with fecundity, neither sperm concentration, morphology, nor motility could be considered diagnostic of infertility either alone or in combination [3]. The limitations of semen analysis have been further reinforced by studies showing that 15 % of those with normal semen analyses by WHO criteria have infertility while other men with abnormal semen parameters are fertile and able initiate a pregnancy naturally [4, 5].

In the current era of assisted reproductive techniques where technology can help overcome defects in sperm function, the value of semen analysis has become even more dubious. Initial reports of intracytoplasmic sperm injection (ICSI) hailed its ability to bypass the natural selection process and enable men with severely impaired semen parameters to achieve both clinical pregnancy and live birth. In his initial paper, Palermo reported that neither sperm concentration, progressive motility, nor morphology had any impact on ICSI outcomes [6]. Further case series corroborated Palermo’s early findings, with studies reporting that neither oligospermia, asthenoozospermia, teratoozospermia, nor oligoasthenoteratoozospermia had any impact on fertilization or pregnancy rates in ICSI [7–10]. The only sperm parameter that appeared to have a negative impact on ICSI outcomes was the use of a totally immotile, or presumably dead, spermatozoon [8]. However, more recent experience has started to suggest that ICSI may not be as effective in some cases with impaired semen parameters, with case series reporting significant deleterious effects on clinical pregnancy rates with ICSI for men with severe asthenoozospermia [11], teratoozospermia [12], and cryptoozospermia [13]. The conflicting evidence on the ability of semen analysis to predict ICSI outcomes reinforces the inadequacy of semen analysis as a measure of male subfertility and stresses the need for a more robust and sophisticated marker for male subfertility.

### Sperm DNA damage

The limitations of semen analysis have led to the investigation of sperm DNA damage as a potential marker for male subfertility. The packaging of DNA within the sperm head is the result of a complicated process requiring extensive compaction and remodeling of the chromatin. Unlike in somatic cells, where DNA is complexed with histones into organizational units called nucleosomes, the DNA in sperm cells is disassembled from the nucleosomal structure with the somatic nucleosomal histones replaced by small basic proteins called protamines during spermiogenesis [14]. As hypothesized by Aitken, while fully protaminated sperm DNA is highly stable and resistant to damage, deficiencies in protamination leave the DNA poorly compacted and more prone to damage [15]. During the chromatin packing process, single-strand and double-strand breaks are naturally induced to allow unwinding of the nucleosomal structure and again to avoid supercoiling. However, these strand breaks are thought to be repaired to prevent the persistence of DNA damage in mature spermatozoa. Defects that affect DNA repair during DNA compaction and packaging may also contribute to sperm DNA damage.

Incomplete apoptosis is thought to be another potential etiology for sperm DNA damage [16]. As in somatic cells, abnormal sperm are programmed to undergo apoptosis. However, because they are transcriptionally and translationally inert, the apoptotic pathways do not continue to completion. The initiated apoptotic process causes the release of reactive oxygen species (ROS) and the induction of sperm DNA damage, resulting in the release of sperm with elevated levels of DNA damage [16].

The post-testicular environment may also play a role in sperm DNA damage, potentially through the action of ROS [15]. Studies have found increased levels of sperm DNA damage in men with longer abstinence periods while other studies have found higher levels of sperm DNA damage in ejaculated sperm when compared to testicular sperm [17]. Animal studies have also suggested a role for the post-testicular environment in sperm DNA damage. Sugunuma et al. used a well-characterized murine model for abnormal spermatogenesis to investigate the origin of sperm DNA damage. He found that in infertile animals, sperm harvested from the cauda epididymis or ejaculate had higher levels of DNA damage and resulted in decreased fertilization rates when compared to sperm harvested from the testicle or caput epididymis, suggesting that DNA damage occurred during epididymal transit [18]. These findings were the first to challenge the paradigm that the epididymal environment protected and promoted the maturation of sperm and suggested that epididymal transit could, in fact, further damage sperm in men with defective spermatogenesis. The contribution of the post-testicular environment to sperm DNA damage has also been demonstrated more recently by Gawecka et al., who showed that luminal fluid within the epididymis and vas deferens can activate sperm chromatin fragmentation in mice [19].

Unfortunately, while significant strides have been made in our understanding of sperm DNA integrity and damage, the exact cause and origin of sperm DNA damage remains unknown.

### Measuring sperm DNA damage

There are a variety of tests available to measure sperm DNA damage, including Comet, sperm chromatin dispersion (SCD or Halo), sperm chromatin structure assay (SCSA), and terminal deoxynucleotidyl transferase-mediated deoxyuridine triphosphate-nick end labeling (TUNEL). While each of the tests assess sperm DNA damage, they are not equivalent, with each analysis measuring a slightly different aspect of DNA damage (Table 1). Indirect tests for sperm DNA damage include SCD and SCSA while Comet and TUNEL are direct tests of sperm DNA breaks.

**Table 1:** Comparison of assays measuring sperm DNA fragmentation

Assay	Method of detection	Advantages	Disadvantages
SCD/Halo [20]	• Sperm loaded in agarose, denatured in acid solution, stained and observed with fluorescence microscopy for chromatin dispersion/halos. • Sperm with nondispersed chromatin (ie small halos) have fragmented DNA. • Interpreted as percentage of sperm with nondispersed chromatin.	• Commercial assay available • Interpretation does not depend on fluorescence or flow cytometry	• Indirect assay only detects ssDNA breaks • Involves acid denaturation
SCSA [21]	• Acid denaturation, then staining with acridine orange and measurement with flow cytometer. • Green-staining sperm have intact DNA while red-staining sperm have denatured DNA. • Interpreted as percentage of red-staining sperm (denatured sperm).	• Has extensive body of literature and established clinical thresholds • Can be performed in fresh or frozen samples	• Proprietary protocol with no commercial assay available • Indirect assay only detects ssDNA breaks • Involves acid denaturation
Comet [26]	• With gel electrophoresis, fragmented DNA forms tail while intact DNA stays in head. • Can be performed in alkaline or neutral conditions. • Tail size correlates to amount of DNA fragmentation within individual spermatozoon. • Interpreted as mean amount of DNA damage per individual spermatozoon.	• Requires small number of sperm cells • Direct assay can examine damage at level of individual spermatozoon • Can detect multiple types of DNA fragmentation	• No established standardized protocol • Time and labour intensive • Requires fresh semen sample
TUNEL [30]	• Labeled nucleotide added to sites of DNA fragmentation with subsequent fluorescence measured by flow cytometry or fluorescence microscopy. • Fluorescent sperm have fragmented DNA. • Interpreted as percentage of fluorescent sperm.	• Direct assay • Commercial assay available • Can be performed in fresh or frozen samples • Detects ssDNA and dsDNA breaks	• No established standardized protocol • Variable clinical thresholds in literature

### Sperm chromatin dispersion (SCD or Halo)

SCD functions on the principle that when sperm are placed in agarose, denatured in acid, and then exposed to a lysing solution to remove DNA-associated proteins, those with intact DNA will disperse around the nucleus (thereby producing a halo around the nucleus) whereas those with fragmented DNA will not disperse. Sperm with fragmented DNA are prone to the induction of single-strand DNA (ssDNA) motifs during the acid denaturation step and give rise to sperm with nondispersed nuclei (very small or absent halos) when viewed under a microscope [20]. The SCD result represents the percentage of sperm present with nondispersed nuclei.

SCD only detects ssDNA motifs created during the acid denaturation step and fails to detect altered bases [20]. While these ssDNA motifs can be created from both single- and double-strand DNA fragments during the acid denaturation step, SCD cannot discriminate the type of DNA fragmentation or quantify the amount of DNA damage at the level of the individual spermatozoon. On the other hand, as an assay that does not depend on colour or fluorescence, SCD is simple, fast, and reliable and does not require an experienced operator to interpret or analyze the results.

### Sperm chromatin structure assay (SCSA)

Like SCD, SCSA also starts with an acid denaturation step and depends on the principle that abnormal DNA is more prone to further fragmentation by acid denaturation than intact DNA. Unlike SCD, SCSA uses a change in fluorescence by acridine orange to differentiate between sperm with fragmented DNA versus those with intact DNA. Acridine orange fluoresces green when bound to double-strand DNA (dsDNA) but changes to red when bound to ssDNA. A flow cytometer is then used to detect the proportion of sperm with green versus red fluorescence and determine the percentage of sperm with fragmented DNA [21].

Like SCD, SCSA can only detect ssDNA motifs created during the acid denaturation step and can neither discriminate the type of DNA fragmentation nor quantify the amount of DNA damage at the level of the individual spermatozoon [21]. Since it requires flow cytometry to detect the change in acridine orange fluorescence from green to red, it is also more expensive and requires experienced operators to conduct the assay and interpret the results.

Since its introduction in 1980, SCSA has been extensively researched, with various studies attempting to define threshold values. Unfortunately, variations in SCSA protocols, artificial reproductive techniques, and patient populations has led to varying thresholds, including 20, 27, and 30 % [22–24]. While there can be considerable intra-individual variability of 30 % in sperm DNA damage as measured by SCSA, clinically meaningful differences that move a patient from likely to achieve pregnancy in IVF to unlikely to achieve pregnancy in IVF occurs in only 11 % of patients [25].

### Comet assay

The Comet assay relies on gel electrophoresis to quantify the amount of DNA damage within an individual spermatozoon. First, the sperm membrane is lysed and the DNA decondensed in a high-concentration salt environment that helps break down disulphide bridges to remove DNA-associated proteins. In an alkaline electrophoretic field, charged broken DNA strands migrate to the cathode, leaving uncharged, unbroken DNA strands behind. The resulting image resembles a comet, with the tail of broken DNA strands trailing away from a head of unbroken DNA strands. A fluorescent dye that binds DNA (such as SYBR green 1) is applied to the slides to enable visualization of the assay by fluorescent microscopy [26].

The Comet assay is able to detect both single- and double-strand DNA breaks as well as altered bases and can detect breaks with both protamine-associated and histone-associated chromatin [26]. Since the Comet assay measures the quantity of DNA damage in an individual spermatozoon, the reported Comet assay results represent the mean damage from groups of individual spermatozoa, with only 50 spermatozoa being needed to obtain a reliable and repeatable result [26]. Compared to other assays, the Comet assay is simple and cheap to perform, but as its interpretation depends on fluorescent microscopy, requires an experienced observer with special equipment to analyze and interpret the slides. Furthermore, the interpretation of numerous spermatozoon Comet assays is a tedious and low-throughput process in which only 600 Comet assays per hour may be assessed manually whereas semi-automated systems can analyze 50 slides per day [26]. However, newer advances have leveraged fully automated processes to improve the speed of Comet analysis by more than 90 %, which may improve the usability of Comet in larger cohorts of patients [27].

Studies done by those currently marketing the Comet assay have attempted to define clinical threshold values for Comet predictive of clinical pregnancy in in vitro fertilization (IVF) and intracytoplasmic sperm injection (ICSI) while assessing the repeatability of the assay [28, 29]. They determined a threshold of 52 % for whole sperm, above which point a pregnancy was unlikely to occur with IVF (OR 76, 95 % CI 8.69–1714, RR 4.75) and at which point they would recommend proceeding to ICSI [29]. In 203 couples undergoing IVF and 136 couples undergoing ICSI, the authors found that live birth rates with IVF fell from 33 % in those with DNA fragmentation <25 to 24 % in those with DNA fragmentation between 25–50 % and to 13 % in those with DNA fragmentation >50 % (*p* = 0.007) [28]. However, there did not appear to be any association between sperm DNA damage as measured by Comet and ICSI outcomes [28]. In their study, they found Comet to be highly repeatable, with a variance of a single assay of 3.73 %, which fell to 2.65 and 2.17 % for duplicate and triplicate assays, respectively [28].

### Terminal deoxynucleotidyl transferase-mediated deoxyuridine triphosphate-nick end labeling (TUNEL)

TUNEL relies on the enzyme terminal deoxynucleotidyl transferase (TdT) to incorporate a biotinylated deoxyuridine triphosphate (dUTP) at the 3′-OH ends found at ssDNA and dsDNA break sites within the sperm DNA. Either flow cytometry or fluorescence microscopy can then be used to assess the fluorescence, with sperm brightness proportion to the level of DNA fragmentation. TUNEL results represent the percentage of sperm with fragmented DNA within the sample [30].

Unlike the previously described indirect tests of sperm DNA damage, TUNEL binds directly to the sites of both ssDNA and dsDNA breaks within the sperm DNA and does not rely on an additional denaturation step to induce breaks in the sperm DNA [30]. However, like the indirect tests, TUNEL cannot discriminate between the types of DNA fragmentation and is not only expensive, but also labour-intensive and requires experienced operators to perform and interpret the results.

Of the tests for sperm DNA damage, TUNEL arguably presents the most potential for variation and is the least standardized. As a result, studies attempting to establish threshold values have resulted in dramatically different values ranging from 4 to 35 % [29].

### The role of sperm DNA fragmentation in IVF and ICSI

Numerous studies have attempted to assess the association between elevated sperm DNA fragmentation and ART outcomes. Unfortunately, variations between sperm DNA fragmentation assays, protocols, and thresholds and differences in study populations have resulted in systematic reviews and meta-analyses fraught with heterogeneity and unable to come to robust conclusions. To some degree, the controversy surrounding sperm DNA fragmentation is expected. If a male factor that adversely affects reproductive outcomes is present alongside a female factor known to have a strong deleterious effect on reproductive outcomes (such as increased female age), then the stronger female factor may obviate or even reverse the measurable adverse impact of sperm DNA fragmentation on reproductive outcomes. Furthermore, many of the studies have grouped patients undergoing IVF and ICSI together despite differences between the two techniques and have examined different clinical endpoints such as clinical pregnancy, miscarriage, and live birth.

The most recent systematic review and meta-analysis investigated 56 studies broken up into IVF (16 studies), ICSI (24 studies), and mixed IVF and ICSI (16 studies) and measuring sperm DNA damage either by SCSA, TUNEL, SCD, or Comet [31]. Overall, they found that sperm DNA damage predicts poor clinical pregnancy rates after IVF and/or ICSI (OR 1.68, 95 % CI 1.49–1.89, *p* < 0.0001). When stratified by type of ART, the impact of sperm DNA damage on clinical pregnancy persisted (OR 1.65, 95 % CI 1.34–2.04, *p* < 0.0001 and OR 1.31, 95 % CI 1.08–1.59, *p* < 0.0068 for IVF and ICSI, respectively) [31]. In keeping with other previously published meta-analyses, this meta-analysis was limited by poorly controlled female factors and a high study heterogeneity (61 %, *p* < 0.001), making it hard to rely on the odds ratios obtained. While acknowledging the limited trustworthiness of their odds ratios, the authors do attempt to explore the clinical relevance of sperm DNA damage in ART and note a greater clinical relevance for sperm DNA in IVF than in ICSI. With a median positive predictive value (PPV) of 79 %, a median negative predictive value (NPV) of 35 %, and a median clinical pregnancy rate of 32 % in IVF, information gleaned from sperm DNA damage can discriminate between expected IVF clinical pregnancy rates of 21 % (positive result) and 35 % (negative result) [31]. On the other hand, from a median PPV of 64 %, a median NPV of 40 %, and a median clinical pregnancy rate of 36 % in ICSI, sperm DNA damage results can only discriminate between expected ICSI clinical pregnancy rates of 36 % (positive result) and 40 % (negative result) [31].

Another recent systematic review and meta-analysis also sought to assess the effect of sperm DNA damage on live birth rates in IVF and ICSI. The authors identified six prospective cohort studies that investigated the impact of sperm DNA damage on live birth rates, with three using SCSA, two using TUNEL, and one using Comet to measure sperm DNA damage. Their meta-analysis for patients undergoing IVF, which comprised four studies with 553 patients, found a significantly higher live birth rate for men with low amount of sperm DNA damage than for those with high levels of sperm DNA damage (RR 1.27, 95 % CI 1.05–1.52, *p* = 0.01) [32]. For patients undergoing ICSI, the authors pooled results from five studies comprising 445 patients and again found a significant increase in live birth rate for men with low levels of sperm DNA damage (RR 1.11, 95 % CI 1.00–1.23, *p* = 0.04) [32]. In an attempt to control for female factors, the authors subsequently conducted a subgroup analysis that included only studies accounting for female factors (age and ovarian reserve). They identified only two studies with a very limited number of treated subjects, but found the impact of sperm DNA damage on IVF live birth rates was amplified significantly (RR 2.76, 95 % CI 1.59–4.80, *p* = 0.0003) whereas the impact of sperm DNA damage on ICSI live birth rates became nonsignificant (RR 1.08, 95 % CI 0.39–2.96) [32]. This systematic review and meta-analysis, which assessed a highly clinically relevant outcome (live birth rate), suggests that higher levels of sperm DNA damage have a significant impact on live birth rates in IVF, but not in ICSI and advocates for ICSI as a potential therapeutic option for men with elevated levels of sperm DNA damage. Unfortunately, one cannot derive a robust conclusion from this systematic review as it is fraught with heterogeneity, with the six studies using both different methods to assess for sperm DNA damage and also different thresholds to define high versus low sperm DNA damage.

Despite the limitations of the aforementioned systematic review, its conclusions agree with some previously published studies. In particular, Zhao et al. conducted a systematic review and meta-analysis to investigate the impact of sperm DNA damage on clinical pregnancy and miscarriage rates in IVF and ICSI. The authors included sixteen cohort studies with 3106 couples undergoing IVF or ICSI and found a significant decrease in pregnancy rates for men with high DNA damage undergoing IVF (OR 0.66, 95 % CI 0.48–0.90, *p* = 0.008) but not for those undergoing ICSI (OR 0.94, 95 % CI 0.70–1.25) [33]. On the other hand, they found a significant increase in miscarriage rates for men with high DNA damage undergoing ICSI (OR 2.68, 95 % CI 1.40–5.14, *p* = 0.003), but not for those undergoing IVF (OR 1.84, 95 % CI 0.98–3.46) [33]. Of note, the authors also stratified their meta-analysis by sperm DNA damage assessment method and found that the different tests for sperm DNA damage behaved differently, with only sperm DNA damage as detected by TUNEL being significantly associated with decreased clinical pregnancy rates while only TUNEL and SCSA were significantly associated with increased miscarriage rates [33]. While this systematic review focused on the surrogate outcomes of clinical pregnancy and miscarriage rates rather than live birth rates, it echoes the review published by Osman et al. in suggesting an impact of sperm DNA damage on clinical pregnancy rates in IVF, but not in ICSI. On the other hand, despite the meta-analysis capturing only 47 miscarriages in the ICSI group and 70 miscarriages in the IVF group, it presents contradictory results showing an association between sperm DNA damage with higher miscarriage rates in ICSI, but not IVF. A further important insight was the indication that the various methods of measuring sperm DNA damage are not equivalent. Again, the results of this systematic review must be interpreted with caution due to heterogeneity, with differing threshold values for high versus low sperm DNA damage being used amongst the included studies and with the review unable to account for female factors.

Older systematic reviews and meta-analyses have looked separately at the impact of sperm DNA damage on miscarriage rates [34, 35] or on clinical pregnancy rates [36]. Unlike Zhao’s meta-analysis, which suggests a different impact for sperm DNA damage on IVF versus ICSI, Robinson [34] and Zini [35] both found that the significant associations between high sperm DNA damage and miscarriage rates did not depend on the method of fertilization used. Collins [36] similarly found a statistically and clinically significant association between sperm DNA damage and clinical pregnancy rates when they pooled data from IVF and ICSI studies. However, when the authors did subgroup analyses of IVF and ICSI studies separately, they were unable to demonstrate any statistically significant impact of sperm DNA damage on clinical pregnancy results [36]. Furthermore, while the effect of sperm DNA damage on reproductive outcomes was statistically significant, but not enough to change the decision to pursue assisted reproduction, they concluded that sperm DNA damage had limited clinical value in the initial evaluation of the infertile male. All three of these older systematic reviews face similar limitations as the more recently published meta-analyses, with a great deal of heterogeneity between the tests of sperm DNA damage used, threshold values for high versus low sperm DNA damage, control of female factors, and the use of surrogate outcomes (like clinical pregnancy) rather than live birth rate. Ultimately, as was noted by the American Society for Reproductive Medicine in 2013, despite the multitude of systematic reviews trying to tease out the impact of sperm DNA damage on outcomes in IVF and ICSI, heterogeneity has prevented any robust conclusions from being drawn on the clinical utility of sperm DNA damage prior to treatment with IVF and ICSI [37].

A potential explanation for the disparate findings seen in contemporary meta-analyses on the impact of sperm DNA damage when assessing clinical pregnancy and miscarriage rates in IVF and ICSI could lie in the difference in sperm selection process between the two methods of fertilization. Importantly, none of the methods of assessing sperm DNA damage permits the evaluation of the individual spermatozoon that goes on to fertilize the oocyte via either method of fertilization. In ICSI, sperm with normal morphology and progressive motility are selected for injection, which may improve fertilization and clinical pregnancy rates when compared to IVF, when fertilization depends on the natural selection of a healthy spermatozoon from a group with a high proportion of abnormal sperm. Studies have shown that in infertile men, morphologically normal motile sperm have higher rates of sperm DNA fragmentation (20–60 %) when compared to those in fertile men [38]. While sperm DNA damage appears to play a lesser role in fertilization and early development than abnormalities in centrosome function or oocyte-activation factor [39], the higher miscarriage rates in ICSI may be suggestive of a late paternal effect of abnormal sperm DNA fragmentation on embryo development [40]. Amongst couples who become pregnant, the natural selection of a spermatozoon in IVF may allow the selection of a near normal spermatozoon with lesser degrees of sperm DNA damage when compared to the artificial selection process used in ICSI, in which the selection of a morphologically normal motile spermotozoon may still mask significant sperm DNA damage that exert their influence not at fertilization, but later during embryonic development [40]. Further evidence of sperm DNA damage representing unmeasurable sperm defects in the entire semen sample rather than simply in the few severely damaged sperm detected in the test result comes from the fact that despite processed semen samples having lower levels of sperm DNA damage than neat (or unprocessed) semen samples, only sperm DNA damage as measured by SCSA in the neat (or unprocessed) semen sample is predictive of reproductive outcomes [41]. On the other hand, when using Comet, sperm DNA damage in both the neat and processed semen samples are predictive of reproductive outcomes [42].

While meta-analyses appear to uniformly suggest a deleterious impact for sperm DNA damage on clinical pregnancy and live birth rates in IVF, they are less clear on the effect of sperm DNA damage on ICSI outcomes. Unfortunately, the number of studies assessing ICSI alone is very limited, which makes it difficult to analyze the true effects of sperm DNA damage on ICSI results. Contemporary studies looking at ICSI results alone have corroborated the adverse effect of impaired sperm DNA integrity on ICSI outcomes, with Esteves et al. showing higher miscarriage and lower live birth rates in couples using ejaculated sperm with high sperm DNA damage (mean DFI 40.9 %) compared to those using testicular sperm with low sperm DNA damage (mean DFI 8.3 %) in a prospective cohort trial of 172 couples [43]. Similarly, Mehta et al. found that 50 % of couples who had previously failed one or more IVF-ICSI cycles using ejaculated sperm with a high sperm DNA damage (mean TUNEL 24.5 %) were able to achieve both pregnancy and live birth with ICSI using testicular sperm with low sperm DNA damage (mean TUNEL 4.6 %) [44]. Taken together, this contemporary data suggests a negative impact for high levels of sperm DNA damage on ICSI results.

### Implementing sperm DNA fragmentation into clinical practice

While the appropriate place for sperm DNA fragmentation in the clinical management of the infertile couple has yet to be clearly elucidated, the growing body of evidence suggests its utility in helping direct the management of the infertile couple. Given the association between sperm DNA fragmentation and early miscarriage in ART, an elevated sperm DNA fragmentation rate in the couple with recurrent ART failure may point to a paternal cause. Even the non-azoospermic couple with recurrent natural pregnancy loss may benefit from investigation into sperm DNA fragmentation that could then predict IVF success and potentially direct a couple to ICSI rather than IVF.

Therapeutic strategies to improve high levels of sperm DNA fragmentation are unfortunately poorly understood. Strategies that have been studied include oral antioxidant medications, varicocele repair, and the use of testicular sperm in ART. Unfortunately, while the evidence does suggest potential benefit to these strategies in the management of elevated sperm DNA fragmentation, the quality of the evidence on the topic is limited by smaller studies.

The seminal paper examining the use of antioxidants to manage elevated sperm DNA fragmentation was performed by Greco et al. in 38 couples who had elevated sperm DNA fragmentation and a failed ICSI cycle attempt. After treatment with 2 months of daily vitamin C and vitamin E, 76 % of men had a decrease in sperm DNA fragmentation from >10 % on TUNEL to <10 % on TUNEL, which resulted in a significantly improved clinical pregnancy rate (48.2 % vs 6.9 %, *p* < 0.05 for post-treatment with antioxidants and pre-treatment with antioxidants, respectively) [45]. While a recent Cochrane review suggested a positive impact of oral antioxidants on live birth rates in couples attending fertility clinics (OR 4.21, 95 % CI 2.08–8.51, *p* < 0.0001), the results were based on a small amount of low quality data [46]. Only two studies included in the analysis looked at sperm DNA fragmentation, but noted an improvement in sperm DNA fragmentation of 13 % with antioxidant therapy [46].

Since oxidative stress is posited as a potential pathophysiological mechanism for varicocele on fertility, varicocele repair has also been touted as a potential strategy to manage elevated sperm DNA fragmentation. Indeed, men with varicoceles appear to have higher levels of sperm DNA fragmentation and varicocele repair is often associated with improvements in sperm DNA fragmentation [47]. However, a meta-analysis revealed that the improvement in sperm DNA fragmentation after varicocele repair was limited to 3.37 % (95 % CI 2.65–4.08 %, *p* < 0.000001), which may not be clinically relevant [48].

The use of testicular sperm in ART for couples with elevated sperm DNA fragmentation has also been investigated. Testicular sperm typically has lower levels of sperm DNA fragmentation when compared to ejaculated sperm [17]. As has been discussed earlier in this review, both Esteves and Mehta have demonstrated improved live birth rates in couples using testicular sperm with lower levels of sperm DNA fragmentation rather than ejaculated sperm for ICSI [43, 44].

In our practice, we test sperm DNA fragmentation in couples who have recurrent pregnancy loss either by natural conception or with ART. Our center typically tests TUNEL and internal testing has shown good concordance of our laboratory’s TUNEL results with reproductive outcomes (unpublished data). Given the large body of evidence behind SCSA, we also use SCSA as an adjunct measure of sperm DNA fragmentation. For patients with elevated levels of sperm DNA fragmentation, we advocate the use of antioxidants and will also proceed with testicular sperm retrieval for use in ICSI for couples with recurrent pregnancy loss using ejaculated sperm with elevated sperm DNA fragmentation.

### Impact of sperm DNA fragmentation on offspring health

Another concern stemming from elevated sperm DNA fragmentation revolves around its impact on offspring. Given the novelty of sperm DNA fragmentation assays and the fact that most ICSI children are only reaching their 20s, the long term impact on offspring remains unknown. Furthermore, ethical issues will likely render high quality comparative studies in humans impossible, currently leaving only evidence from animal models and indirect extrapolations to address the question.

Animal studies in rodents have demonstrated that the ICSI offspring derived from males with elevated sperm DNA fragmentation are prone to abnormal growth and behaviour, premature aging, and the development of tumours during later life [49]. Indirect evidence of the impact of sperm DNA fragmentation on offspring health can be derived from studies linking paternal age and smoking with offspring health. While no studies have looked at the direct association between sperm DNA fragmentation and offspring health in humans, studies have linked both increasing paternal age and smoking with increased sperm DNA fragmentation while other studies have linked these paternal states to increased incidences of childhood cancer, schizophrenia, and neural tube defects in the offspring [50–54].

Ultimately, while current animal models and extrapolative data in human studies raise concern of health problems in the offspring derived from sperm with elevated levels of DNA fragmentation, longitudinal data from current and future ICSI offspring are required to obtain a fuller understanding of the impact of sperm DNA fragmentation on offspring health.

### Next steps and challenges

Our current efforts to understand the clinical utility of sperm DNA damage on reproductive outcomes during IVF and ICSI are currently being hampered by our poor understanding of sperm DNA damage, including the effects of measured sperm DNA damage on other spermatozoa in the semen sample that do not have clinically detectable damage. Zhao’s finding that the different methods of assessing sperm DNA damage behave differently is in agreement with previous studies and highlights a major gap in our knowledge of sperm DNA damage [33–36]. Our current understanding of the clinical implications of sperm DNA damage is at a nascent stage. As mentioned previously, we do not yet definitively know the etiology of sperm DNA damage nor do we know the true clinical implications of the types or degree of sperm DNA damage on reproductive outcomes. Without this knowledge, it is difficult to determine both the best clinical assay for sperm DNA damage and the appropriate thresholds to use in order to standardize our assessment of sperm DNA damage. An increased understanding of the repair mechanisms and capabilities for sperm DNA damage both during spermiogenesis and within the oocyte during fertilization is also needed. Finally, we remain unable to determine the genetic status of the fertilizing spermatozoon and are currently unable to use our current assessments of sperm DNA damage to help select sperm with intact DNA for ICSI. Once we improve our understanding of sperm DNA damage and are able to establish standardized methodologies and thresholds to assess sperm DNA damage, we can then consider undertaking large, prospective, randomized controlled trials to truly assess the impact of sperm DNA damage on reproductive outcomes in IVF and ICSI.

## Conclusion

For many years, semen analysis has been the cornerstone for evaluation of male infertility despite limitations of its utility to predict reproductive outcomes in IVF and ICSI. As a result, assessment of sperm DNA damage has been touted as a useful adjunct to help predict outcomes in IVF and ICSI. Numerous large systematic reviews appear to show relationships between sperm DNA damage and reproductive outcomes in IVF and ICSI, though these reviews are limited by the heterogeneity of the underlying studies that make it difficult to draw definitive conclusions from their analyses. While our understanding of the etiology, measurement, and functional implications of sperm DNA damage remains incomplete, these sperm DNA fragmentation tests provide additional information on the critical role of sperm in the development and maintenance of a pregnancy. However, at this time, there is not yet definitive evidence to support the routine and widespread use of sperm DNA damage testing in general reproductive practice.

## Abbreviations

dsDNA: Double-strand DNA
dUTP: Deoxyuridine triphosphate
ICSI: Intracytoplasmic sperm injection
IVF: In vitro fertilization
LIFE: Longitudinal Investigation of Fertility and the Environment
OR: Odds ratio
ROS: Reactive oxygen species
RR: Risk ratio
SCD: Sperm chromatin dispersion
SCSA: Sperm chromatin structure assay
ssDNA: Single-strand DNA
Tdt: Terminal deoxynucleotidyl transferase
TTP: Time-to-pregnancy
TUNEL: Terminal deoxynucleotidyl transferase-mediated deoxyuridine triphosphate-nick end labeling
WHO: World Health Organization

## References

[1] Cooper TG, Noonan E, von Eckardstein S (2010). World health organization reference values for human semen characteristics. Hum Reprod Update.

[2] Buck Louis GM, Sundaram R, Schisterman EF (2014). Semen quality and time to pregnancy: the longitudinal investigation of fertility and the environment study. Fertil Steril.

[3] Guzick DS, Overstreet JW, Factor-Litvak P, et al. Sperm morphology, motility, and concentration in fertile and infertile men. N Engl J Med. 2001;345(19):1388–93. http://www.nejm.org/doi/full/10.1056/NEJMoa003005. 10.1056/NEJMoa003005 11794171

[4] Nallella KP, Sharma RK, Aziz N, Agarwal A (2006). Significance of sperm characteristics in the evaluation of male infertility. Fertil Steril.

[5] Agarwal A, Allamaneni SS (2005). Sperm DNA damage assessment: a test whose time has come. Fertil Steril.

[6] Palermo G, Joris H, Derde MP, Camus M, Devroey P, Van Steirteghem A (1993). Sperm characteristics and outcome of human assisted fertilization by subzonal insemination and intracytoplasmic sperm injection. Fertil Steril.

[7] Mansour RT, Aboulghar MA, Serour GI, Amin YM, Ramzi AM (1995). The effect of sperm parameters on the outcome of intracytoplasmic sperm injection. Fertil Steril.

[8] Nagy ZP, Liu J, Joris H (1995). The result of intracytoplasmic sperm injection is not related to any of the three basic sperm parameters. Hum Reprod.

[9] Nijs M, Vanderzwalmen P, Vandamme B (1996). Fertilizing ability of immotile spermatozoa after intracytoplasmic sperm injection. Hum Reprod.

[10] Vandervorst M, Tournaye H, Camus M, Nagy ZP, Van Steirteghem A, Devroey P (1997). Patients with absolutely immotile spermatozoa and intracytoplasmic sperm injection. Hum Reprod.

[11] Mitchell V, Rives N, Albert M (2006). Outcome of ICSI with ejaculated spermatozoa in a series of men with distinct ultrastructural flagellar abnormalities. Hum Reprod.

[12] De Vos A, Van De Velde H, Joris H, Verheyen G, Devroey P, Van Steirteghem A (2003). Influence of individual sperm morphology on fertilization, embryo morphology, and pregnancy outcome of intracytoplasmic sperm injection. Fertil Steril.

[13] Strassburger D, Friedler S, Raziel A, Schachter M, Kasterstein E, Ron-el R (2000). Very low sperm count affects the result of intracytoplasmic sperm injection. J Assist Reprod Genet.

[14] Aitken RJ, De Iuliis GN, McLachlan RI (2009). Biological and clinical significance of DNA damage in the male germ line. Int J Androl.

[15] Aitken RJ, Bronson R, Smith TB, De Iuliis GN (2013). The source and significance of DNA damage in human spermatozoa; a commentary on diagnostic strategies and straw man fallacies. Mol Hum Reprod.

[16] Aitken RJ, De Iuliis GN (2010). On the possible origins of DNA damage in human spermatozoa. Mol Hum Reprod.

[17] Greco E, Scarselli F, Iacobelli M (2005). Efficient treatment of infertility due to sperm DNA damage by ICSI with testicular spermatozoa. Hum Reprod.

[18] Suganuma R, Yanagimachi R, Meistrich ML (2005). Decline in fertility of mouse sperm with abnormal chromatin during epididymal passage as revealed by ICSI. Hum Reprod.

[19] Gawecka JE, Boaz S, Kasperson K, Nguyen H, Evenson DP, Ward WS (2015). Luminal fluid of epididymis and vas deferens contributes to sperm chromatin fragmentation. Hum Reprod.

[20] Fernandez JL, Muriel L, Goyanes V (2005). Simple determination of human sperm DNA fragmentation with an improved sperm chromatin dispersion test. Fertil Steril.

[21] Evenson DP (2013). Sperm chromatin structure assay (SCSA(R)). Methods Mol Biol.

[22] Boe-Hansen GB, Fedder J, Ersboll AK, Christensen P (2006). The sperm chromatin structure assay as a diagnostic tool in the human fertility clinic. Hum Reprod.

[23] Larson KL, DeJonge CJ, Barnes AM, Jost LK, Evenson DP (2000). Sperm chromatin structure assay parameters as predictors of failed pregnancy following assisted reproductive techniques. Hum Reprod.

[24] Virro MR, Larson-Cook KL, Evenson DP (2004). Sperm chromatin structure assay (SCSA) parameters are related to fertilization, blastocyst development, and ongoing pregnancy in in vitro fertilization and intracytoplasmic sperm injection cycles. Fertil Steril.

[25] Oleszczuk K, Giwercman A, Bungum M (2011). Intra-individual variation of the sperm chromatin structure assay DNA fragmentation index in men from infertile couples. Hum Reprod.

[26] Olive PL, Banath JP (2006). The comet assay: a method to measure DNA damage in individual cells. Nat Protoc.

[27] Albert O, Reintsch WE, Chan P, Robaire B (2016). HT-COMET: a novel automated approach for high throughput assessment of human sperm chromatin quality. Hum Reprod.

[28] Simon L, Proutski I, Stevenson M (2013). Sperm DNA damage has a negative association with live-birth rates after IVF. Reprod Biomed Online.

[29] Simon L, Lutton D, McManus J, Lewis SE (2011). Sperm DNA damage measured by the alkaline comet assay as an independent predictor of male infertility and in vitro fertilization success. Fertil Steril.

[30] Sharma R, Masaki J, Agarwal A (2013). Sperm DNA fragmentation analysis using the TUNEL assay. Methods Mol Biol.

[31] Simon L, Zini A, Dyachenko A, Ciampi A, Carrell DT. A systematic review and meta-analysis to determine the effect of sperm DNA damage on in vitro fertilization and intracytoplasmic sperm injection outcome. Asian J Androl. 2016. doi: 10.4103/1008-682X.182822. 10.4103/1008-682X.182822 PMC5227680 27345006

[32] Osman A, Alsomait H, Seshadri S, El-Toukhy T, Khalaf Y (2015). The effect of sperm DNA fragmentation on live birth rate after IVF or ICSI: A systematic review and meta-analysis. Reprod Biomed Online.

[33] Zhao J, Zhang Q, Wang Y, Li Y (2014). Whether sperm deoxyribonucleic acid fragmentation has an effect on pregnancy and miscarriage after in vitro fertilization/intracytoplasmic sperm injection: a systematic review and meta-analysis. Fertil Steril.

[34] Robinson L, Gallos ID, Conner SJ (2012). The effect of sperm DNA fragmentation on miscarriage rates: a systematic review and meta-analysis. Hum Reprod.

[35] Zini A, Boman JM, Belzile E, Ciampi A (2008). Sperm DNA damage is associated with an increased risk of pregnancy loss after IVF and ICSI: Systematic review and meta-analysis. Hum Reprod.

[36] Collins JA, Barnhart KT, Schlegel PN (2008). Do sperm DNA integrity tests predict pregnancy with in vitro fertilization?. Fertil Steril.

[37] Practice Committee of the American Society for Reproductive Medicine (2013). The clinical utility of sperm DNA integrity testing: a guideline. Fertil Steril.

[38] Avendano C, Franchi A, Taylor S, Morshedi M, Bocca S, Oehninger S (2009). Fragmentation of DNA in morphologically normal human spermatozoa. Fertil Steril.

[39] Tesarik J, Mendoza C, Greco E (2002). Paternal effects acting during the first cell cycle of human preimplantation development after ICSI. Hum Reprod.

[40] Tesarik J, Greco E, Mendoza C (2004). Late, but not early, paternal effect on human embryo development is related to sperm DNA fragmentation. Hum Reprod.

[41] Bungum M, Spano M, Humaidan P, Eleuteri P, Rescia M, Giwercman A (2008). Sperm chromatin structure assay parameters measured after density gradient centrifugation are not predictive for the outcome of ART. Hum Reprod.

[42] Simon L, Brunborg G, Stevenson M, Lutton D, McManus J, Lewis SE (2010). Clinical significance of sperm DNA damage in assisted reproduction outcome. Hum Reprod.

[43] Esteves SC, Sánchez-Martín F, Sánchez-Martín P, Schneider DT, Gosálvez J. Comparison of reproductive outcome in oligozoospermic men with high sperm DNA fragmentation undergoing intracytoplasmic sperm injection with ejaculated and testicular sperm. Fertil Steril. 2015;104(6):1398–405. http://www.fertstert.org/article/S0015-0282(15)01874-9/abstract. 10.1016/j.fertnstert.2015.08.028 26428305

[44] Mehta A, Bolyakov A, Schlegel PN, Paduch DA (2015). Higher pregnancy rates using testicular sperm in men with severe oligospermia. Fertil Steril.

[45] Greco E, Romano S, Iacobelli M (2005). ICSI in cases of sperm DNA damage: beneficial effect of oral antioxidant treatment. Hum Reprod.

[46] Showell MG, Mackenzie-Proctor R, Brown J, Yazdani A, Stankiewicz MT, Hart RJ. Antioxidants for male subfertility. Cochrane Database Syst Rev. 2014;(12):CD007411. doi: 10.1002/14651858.CD007411.pub3. 10.1002/14651858.CD007411.pub3 25504418

[47] Zini A, Dohle G (2011). Are varicoceles associated with increased deoxyribonucleic acid fragmentation?. Fertil Steril.

[48] Wang YJ, Zhang RQ, Lin YJ, Zhang RG, Zhang WL (2012). Relationship between varicocele and sperm DNA damage and the effect of varicocele repair: a meta-analysis. Reprod Biomed Online.

[49] Fernandez-Gonzalez R, Moreira PN, Perez-Crespo M (2008). Long-term effects of mouse intracytoplasmic sperm injection with DNA-fragmented sperm on health and behavior of adult offspring. Biol Reprod.

[50] Fraga CG, Motchnik PA, Wyrobek AJ, Rempel DM, Ames BN (1996). Smoking and low antioxidant levels increase oxidative damage to sperm DNA. Mutat Res.

[51] Schmid TE, Eskenazi B, Baumgartner A (2007). The effects of male age on sperm DNA damage in healthy non-smokers. Hum Reprod.

[52] Ji BT, Shu XO, Linet MS (1997). Paternal cigarette smoking and the risk of childhood cancer among offspring of nonsmoking mothers. J Natl Cancer Inst.

[53] Sipos A, Rasmussen F, Harrison G (2004). Paternal age and schizophrenia: a population based cohort study. BMJ.

[54] McIntosh GC, Olshan AF, Baird PA (1995). Paternal age and the risk of birth defects in offspring. Epidemiology.

